# Ambulatory healthcare for patients with depression: an integration perspective/La atención ambulatoria a los pacientes con depresión: una perspectiva integradora

**Published:** 2012-05-29

**Authors:** A. Retolaza Balsategui, C. Calderón Gómez, J. Payo Gordon, E. Zallo Atxútegui, A. Bacigalupe de la Hera, I. Mosquera Metcalfe

**Affiliations:** Head of Clinical Management Unit (UGC, Unidad de Gestión Clínica), Bizkaia Mental Health Services, Bilbao, Basque Country, Spain; GP, Alza Health Centre, Donostia-San Sebastián, Basque Country, Spain; Primary Care Research Unit, Bilbao, Basque Country, Spain; Bizkaia Mental Health Services, Bilbao, Basque Country, Spain; Department of Health and Consumer Affairs, Vitoria-Gasteiz, Basque Country, Spain; Primary Care Research Unit, Gipuzkoa, Donostia-San Sebastián, Basque Country, Spain

**Keywords:** depression, qualitative research, collaborative care, depresión, investigación cualitativa, atención colaborativa

## Introduction

Numerous studies [1–3] have indicated increasing rates of depression detected in specialised consultations (SC) and primary care (PC). To tackle this problem, the WHO suggests an integrated approach to care [4] and this represents a great challenge. There is currently a debate on new models of healthcare management (such as those focusing on chronic disease and collaborative care) and it seems necessary to investigate various alternatives. Collaborative interventions are effective [5], but little is known about which of their characteristics are essential for them to be successful [6]. Understanding this is important since they are complex and more costly than traditional interventions. In particular, in order to undertake such interventions successfully, we need to assess the attitudes and approaches of all those involved.

In relation to this, our team conducted a research study [7] with general practitioners (GPs) and psychiatrists in the public health system, the results of which confirmed the importance of and current deficiencies in links between these professionals, as well as the need for patient-focused models of collaboration and care. After this work, we concluded that it was necessary to widen our vision of the problem by collecting data on the perspective of other stakeholders.

With this objective, we carried out a second project in which we analysed: on the one hand, the perceptions of other professionals involved (psychologists, specialised nurses and social workers), who we had not been able to approach in the previous study; and, on the other, the opinions of patients themselves, those diagnosed and treated for depression, about the care they had received.

## Methods

### Analysis

The methodology was qualitative, based on discussion groups and interviews recorded, with prior consent, and subsequently transcribed. We then applied a model of sociological discourse analysis using MAXQDA software for text analysis and subsequent validation, by triangulation between researchers and comparison with participants.

### Setting and subjects

Bilbao Health Region, Basque Health Service-Osakidetza. Centres and patients were sampled by socioeconomic (SE) level: high vs. low, using open, flexible purposive sampling, with various groups for each modality of care in order to achieve a higher degree of saturation.

### Study 1

Six discussion groups were organised, four with GPs (n=29) and two with psychiatrists (n=13). The patient’s local health centre was grouped according the SE level (high or low).

### Study 2

A further six discussion groups were organised: 1) two with patients seen in PC (one corresponding to high SE level and one to low SE level); 2) another two with patients seen in SC, also grouped according to SE level; 3) a group of psychologists from various different mental health centres (MHC); and 4) a mixed group of nurses and social workers from SC.

## Results

The views of GPs and psychiatrists differ as a function of their place of work within the health care system. The type of relationship with patients, the lack of accuracy of diagnoses of depression, professional self-perceptions, and a lack of mutual knowledge between the different levels of care determine the clinical process. Health and social contexts are essential for interpreting the views of professionals with respect to their patients.

**Figure 1.**
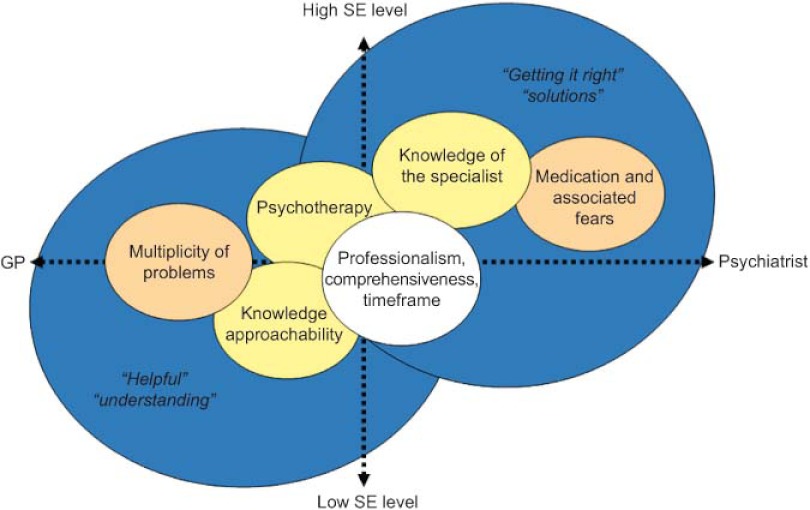
Patients’ expectations and assessment.

Patients follow a variety of different pathways through health services, including PC and SC as well as private care. Expectations and views of the care received also vary widely. Some patients seek support and understanding and appreciate the knowledge and approachability of their GP. Those who value specialised care more are seeking to resolve their symptoms. Such patients tend to have a higher SE level (Figure 1).

Psychologists, specialised nurses and social workers stress their role in listening and giving support. Professionals believe that there is a high degree of medicalisation of life and/or social problems.

## Discussion

Despite advances in recent years, there remains a high degree of uncertainty about which are the most effective practices to adopt for patients with mental health problems and how they should be organised at the various levels of care. This uncertainty leads to excess variability in clinical practice, which is influenced by organisational dynamics closely related to the characteristics and degree of development of each health system [8–10].

Despite the importance of the subject, little research has been carried out on the views of the various stakeholders involved in demand, diagnosis and treatment processes in mental illness. In particular, we need to deepen our understanding of patient perceptions and the way that these are expressed [11, 12]. Many studies published in recent years endorse the need for qualitative research in this area [13–15].

## Conclusions

The provision of healthcare to people diagnosed with depression requires patient-centred approaches shared between professionals. Management of these patients is influenced by the cultural and organisational context in which they are seen. Accordingly, we need to improve our understanding of the specific circumstances at each level of care. Further, coordination between services and levels of care must be promoted through active health policies, and programmes for professional training should be tailored to meet the real needs of professionals and patients.

Currently our team is carrying out a systematic review of the scientific literature to identify the best evidence on models for collaborative work and their contexts. Our aim is to produce an interprofessional consensus document with the strategies to follow to provide effective care for patients diagnosed with depression in our area [16]. Once the evidence gathered, we will organise various working sessions, both face-to-face and over the internet, with the participation of psychiatrists, GPs and other professionals involved in this care area.

Once consensus is reached, we will set up an intervention and evaluation pilot project of an integrated care model in our area. This will enable us to put into practice the results of our research work and give continuity to a participatory research approach that encourages the involvement of stakeholders. In this project, we will use participatory action research methods [17] and it is envisaged that two teams of mental health and PC professionals from the provinces of Gipuzkoa and Bizkaia will participate.

## Conference abstract Spanish

## Introducción

Múltiples estudios [1–3] señalan la creciente presencia de la depresión en consultas especializadas (AE) y de atención primaria (AP). Para enfrentar el problema la OMS sugiere un abordaje asistencial integrado [4]. Ello supone un importante desafío. Asistimos a un debate sobre nuevos modelos de gestión (enfermedades crónicas, atención colaboradora,…) y parece necesario investigar diferentes alternativas. Las intervenciones colaborativas son efectivas [5], pero se conoce poco sobre los aspectos de las mismas que resultan esenciales [6]. Este conocimiento resulta clave a la hora de implantarlas, ya que son complejas y más costosas que las tradicionales. Para desarrollarlas con éxito necesitamos explorar actitudes y planteamientos de los diversos actores implicados.

Respecto a esta problemática, nuestro equipo ha llevado a cabo una investigación [7] con médicos de familia y psiquiatras de nuestro sistema público de salud, cuyos resultados confirman la importancia y las deficiencias de sus vínculos, así como la necesidad de modelos de colaboración y de enfoques asistenciales centrados en el paciente. Tras este trabajo concluímos que precisábamos ampliar nuestra perspectiva del problema recogiendo la visión de otros agentes del proceso.

Con ese objetivo llevamos a cabo un segundo proyecto en el que pudimos analizar, por un parte, las percepciones de otros profesionales involucrados (psicólogos, enfermería especializada y trabajadoras sociales), a quienes no habíamos podido acceder en el estudio anterior. Y sobre todo, dicho proyecto nos permitió investigar las valoraciones de los propios pacientes diagnosticados y tratados por depresión acerca de la asistencia recibida.

## Métodos

### Análisis

Metodología cualitativa. Grupos de discusión. Entrevistas grabadas previa autorización y posteriormente transcritas. Modelo de análisis sociológico del discurso con ayuda del programa MAXqda para el tratamiento de los textos. Posterior validación mediante triangulación entre los investigadores y contraste con los participantes.

### Ámbito y sujetos de estudio

Área Sanitaria de Bizkaia. Servicio Vasco de Salud-Osakidetza. Muestra de centros y pacientes según nivel Socio-Económico (SE) alto vs. bajo. Muestreo intencional abierto y flexible partiendo de varios grupos por cada modalidad de atención con objeto de conseguir mayor grado de saturación.

### Estudio 1

Se organizaron 4 Grupos de discusión con médicos de AP (n=29) y 2 con psiquiatras (n=13). Los centros de referencia se clasificaron en dos tipos según SE alto o bajo.

### Estudio 2

Se organizaron 6 grupos de discusión: 1) Dos con pacientes atendidos en AP (uno de SE alto y otro bajo); 2) Otros dos con pacientes atendidos en AE, igualmente clasificados según SE; 3) Un grupo de psicólogos de distintos Centros de Salud Mental (CSM); 4) Un grupo mixto con diplomados en enfermería y trabajadoras sociales de AE.

## Resultados

Las perspectivas de médicos de familia y psiquiatras difieren en relación con su ubicación en el sistema asistencial. El tipo de vinculación con el paciente, la imprecisión del diagnóstico de depresión, las autopercepciones profesionales y las carencias en el conocimiento entre niveles determinan el proceso clínico. Los contextos sanitario y social resultan esenciales para interpretar las visiones de los profesionales respecto a los pacientes.

**Figura 2.**
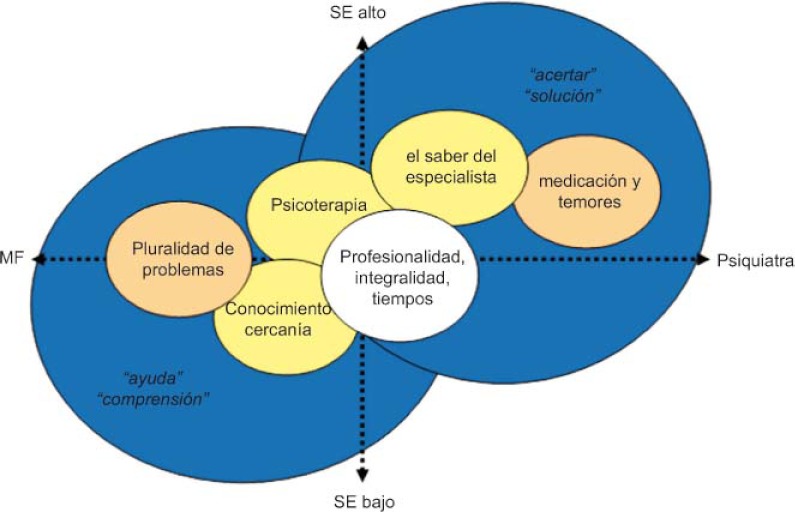
Expectativas y valoraciones de los pacientes.

Entre éstos existen diversidad de trayectorias por los servicios de salud, incluyendo tanto a AP como AE y tratamientos privados. También hay diversas expectativas y valoraciones de la atención recibida. Algunos pacientes buscan apoyo y comprensión apreciando el conocimiento y cercanía que les proporciona su médico de familia. Los que valoran más la atención especializada buscan acierto en la solución de sus síntomas. Esta última situación tiende a observarse con más frecuencia en personas de mejor nivel SE (Figura 2).

Psicólogos, enfermería especializada y trabajadoras sociales destacan su rol de apoyo y escucha. Los profesionales encuentran que existe una importante medicalización de problemas vitales y/o sociales.

## Discusión

A pesar de los avances logrados en los últimos años, existe incertidumbre sobre cuáles son las prácticas más eficaces a desplegar y cómo organizarlas en los diferentes niveles de atención. Estas carencias se traducen en una excesiva variabilidad clínica que procede de dinámicas organizativas estrechamente vinculadas a las características y evolución de los distintos sistemas sanitarios [8–10].

A pesar de la relevancia del tema son escasas las investigaciones que se interrogan sobre las percepciones de los diversos agentes en los procesos de demanda, diagnóstico y tratamiento de los trastornos mentales. De manera especial necesitamos seguir profundizando en el conocimiento de estas percepciones y en la forma en que se manifiestan en los pacientes [11, 12]. Abundantes trabajos publicados en los últimos años avalan la pertinencia de la investigación cualitativa en este campo [13–15].

## Conclusiones

La asistencia a las personas diagnosticadas de depresión requiere aproximaciones centradas en el paciente y compartidas por los profesionales. El manejo de los pacientes es modulado por el contexto cultural y organizativo en el que son atendidos. Se necesita mejor conocimiento sobre las circunstancias concretas que operan en cada nivel asistencial. La coordinación entre servicios y niveles de atención debe ser promovida desde políticas sanitarias activas. Los programas de entrenamiento profesional deben adaptarse a las necesidades reales de profesionales y pacientes.

Actualmente nuestro equipo está llevado a cabo una revisión sistemática de la literatura científica a fin de encontrar la mejor evidencia posible sobre modelos de trabajo colaborativo y sus contextos. Pretendemos elaborar un documento de consenso interprofesional con las estrategias a seguir para lograr una atención efectiva a los pacientes diagnosticados de depresión en nuestro medio [16]. A partir de la evidencia hallada organizaremos varias sesiones de trabajo, tanto presencial como a través de Internet, en las que participarán psiquiatras, médicos de familia y otros profesionales involucrados en tareas asistenciales.

Con el resultado de este consenso se llevará a cabo una experiencia piloto para la intervención y evaluación de un modelo de atención integrada en nuestro medio. Dicho proyecto permitirá poner en práctica los resultados de nuestra línea de trabajo y dar continuidad a un enfoque participativo de investigación que favorezca el protagonismo de los agentes implicados. En este trabajo utilizaremos metodología de investigación-acción-participación [17]. Está previsto que participen dos equipos de salud mental y AP en Gipuzkoa y Bizkaia.
